# Determination of circulating *Mycobacterium tuberculosis *strains and transmission patterns among pulmonary TB patients in Kawempe municipality, Uganda, using MIRU-VNTR

**DOI:** 10.1186/1756-0500-4-280

**Published:** 2011-08-11

**Authors:** Lydia Nabyonga, David P Kateete, Fred A Katabazi, Paul R Odong, Christopher C Whalen, Katherine R Dickman, Joloba L Moses

**Affiliations:** 1 Department of Medical Microbiology, School of Biomedical Sciences, Makerere University College of Health Sciences, Kampala, Uganda; 2 Epidemiology and Biostatistics, College of Public Health, University of Georgia, Athens, USA; 3 Department of Pediatrics, Boston Medical Center, Boston University, Boston, USA

## Abstract

**Background:**

Mycobacterial interspersed repetitive units - variable number of tandem repeats (MIRU-VNTR) genotyping is a powerful tool for unraveling clonally complex *Mycobacterium tuberculosis *(MTB) strains and detection of transmission patterns. Using MIRU-VNTR, MTB genotypes and their transmission patterns among patients with new and active pulmonary tuberculosis (PTB) in Kawempe municipality in Kampala, Uganda was determined.

**Results:**

MIRU-VNTR genotyping was performed by PCR-amplification of 15 MTB-MIRU loci from 113 cultured specimens from 113 PTB patients (one culture sample per patient). To determine lineages, the genotypes were entered into the MIRU-VNTR*plus *database [http://www.miru-vntrplus.org/] as numerical codes corresponding to the number of alleles at each locus. Ten different lineages were obtained: Uganda II (40% of specimens), Uganda I (14%), LAM (6%), Delhi/CAS (3%), Haarlem (3%), Beijing (3%), Cameroon (3%), EAI (2%), TUR (2%) and S (1%). Uganda I and Uganda II were the most predominant genotypes. Genotypes for 29 isolates (26%) did not match any strain in the database and were considered unique. There was high diversity of MIRU-VNTR genotypes, with a total of 94 distinct patterns. Thirty four isolates grouped into 15 distinct clusters each with two to four isolates. Eight households had similar MTB strains for both index and contact cases, indicating possible transmission.

**Conclusion:**

MIRU-VNTR genotyping revealed high MTB strain diversity with low clustering in Kawempe municipality. The technique has a high discriminatory power for genotyping MTB strains in Uganda.

## Background

Tuberculosis (TB) is a leading cause of morbidity and mortality throughout sub-Saharan Africa, and Uganda ranks sixteenth among countries with the highest burden of disease [1]. Co-infection with HIV/AIDS and the emergence of multi-drug resistant (MDR) *Mycobacterium tuberculosis *(MTB) strains have made TB a major public health problem [2]. The incidence of TB in Uganda is estimated at 330 cases per 100,000 persons per year, and this includes both HIV infected and non-HIV infected patients [3]. TB prevalence in Uganda is believed to be higher than reported due to lack of sufficient healthcare; indeed, many people are not aware that they are infected with MTB and this has led to low levels of diagnosis and treatment [4]. Uganda also has one of the lowest TB cure rates (32%) and high drug default rate [1], which may lead to an increase in drug resistance mutations.

Molecular genotyping tools for MTB such as *IS*6110-based restriction fragment length polymorphism (RFLP), "regions of difference" (RD) analysis, spoligotyping, MIRU-VNTR, and single nucleotide polymorphism (SNP) analysis have become invaluable in TB diagnosis and investigations of disease transmission dynamics, outbreaks and phylogenetics [5,6]. Of the MTB genotyping tools, the gold standard is *IS*6110-RFLP, a laborious method that requires large amounts of DNA per isolate, and has poor inter-laboratory reproducibility [7]. MIRU-VNTR, a faster genotyping method with discriminatory power higher than that of *IS*6110 RFLP, has recently been introduced [7,8]. MIRU-VNTR genotyping is performed by amplifying a panel of 12, 15 or 24 loci [9]; genotyping with a panel of 15 loci is best suited for epidemiologic studies [7,10]. Additionally, MIRU-VNTR can detect mixed MTB strains in a single sputum sample [7,11]. Due to its portable data format, MIRU-VNTR can be used as a multi-purpose tool for strain identification using a reference database [2]. However, the choice of appropriate loci for MIRU-VNTR requires evaluation in diverse MTB lineages in countries with high TB prevalence [7]. Mixed MTB infections in Ugandan patients with pulmonary TB (PTB) were recently reported using MIRU-VNTR [11], but genotypes/strains for the entire patient population were not determined. In this study, we aimed to determine the distribution and diversity of MTB lineages in Kawempe municipality using MIRU-VNTR genotyping, and assess the ability of the technique to discriminate the predominant genotypes and detect transmission in this community.

## Methods

### Patients, sample processing and cultures

This study was approved by the Joint Clinical Research Centre (JCRC) Institutional Review Board (Kampala, Uganda) and the University Hospitals Cleveland Institutional Review Board (Cleveland, Ohio). Informed written consent was obtained from patients who participated. Sputum samples were collected from patients with at least one positive culture for MTB, who were previously enrolled in the Kawempe Community Household Contact Study, an ongoing epidemiological study in Kampala, Uganda, from which several papers have been published [12-15]. Samples were collected consecutively from October 2007 through February 2009, from patients with PTB symptoms who reported not having received treatment for TB in the preceding month. Patient demographics, sample processing and cultures, drug susceptibility testing and DNA extraction are described in Dickman et al, 2010 [11]. Cultures were confirmed as MTB by PCR-detection of a 500 bp fragment of the *IS*6110, which is common in the members of the MTB complex [16].

### MIRU-VNTR PCR and data analysis

MIRU-VNTR genotyping was performed by PCR-amplification of a panel of 15 MTB MIRU loci using primers described in the MIRU-VNTR standard protocol [8,11,17]. To size amplicons, gel (3% agarose in TBE) electrophoresis for three hours at 120 constant voltage was performed. The allele calling table in the Supply protocol [8,17] was used to assign the number of alleles corresponding to the amplicon sizes. To determine MTB strain lineages, relatedness or clustering, the MIRU-VNTR genotypes (see additional file 1) were matched with reference strains in the MIRU-VNTR*plus *database (http://www.miru-vntrplus.org/), using a categorical coefficient of 1 and a distance cut off of < 0.3 that corresponds to a seven locus difference. Then, a Neighbor Joining dendrogram was constructed from the strains' genotypes using the MIRU-VNTRplus online program, on assumption of different evolutionary rates (molecular clocks) for MTB MIRU loci [9].

A cluster was defined as two or more patients' strains with identical genetic patterns. Clusters were assumed to have arisen from recent transmission, and the clustering rate was used to determine recent transmission of MTB [18]. The minimum estimate of the proportion of TB cases related to recent transmission was calculated using the formula: (number of clustered patients - number of clusters)/total number of patients [18]. To determine the discriminatory power of MIRU-VNTR for this patient population, the Hunter Gaston Discriminatory Index (HGDI) [19] was calculated.

## Results and Discussion

### High strain diversity in Kawempe municipality

Between October 2007 and May 2009, 113 MTB cultures from 113 patients with PTB were genotyped with MIRU-VNTR using a panel of 15 loci [11]. Ten distinct strains were identified; EAI, Delhi/CAS, Uganda I, Uganda II, LAM, Haarlem, S, Beijing, Cameroon and TUR. Isolates from 29 (26%) patients did not match any strain in the database and were regarded unique (see Table 1). These findings agree with a previous study [20], which found strains of different lineages including Delhi/CAS, LAM and Beijing in Rubaga municipality, Uganda, using RD genotyping. Furthermore, in this study, Uganda I and Uganda II were the predominant lineages (at 14% and 40% prevalence, respectively), followed by LAM (5%). An earlier study using RD genotyping reported MTB "Uganda genotype" as the predominant strain in Rubaga municipality [21]. The Beijing, Dehli/CAS, Haarlem and Cameroon strains were individually found in only 3% of the patients. One MDR strain was unique while two with mono-resistance to isoniazid were of EAI lineage. Another strain with mono-resistance to streptomycin was unique.

**Table 1:** Distribution of MTB lineages

Patients, N = 113	
**Lineage/strain**	**Number (%)**

**Uganda I**	**16 (14)**

**Uganda II**	**45 (40)**

LAM	6 (5)

Beijing	3 (3)

Dehli/CAS	3 (3)

Haarlem	3 (3)

Cameroon	3 (3)

TUR	2 (2)

EAI	2 (2)

S	1 (1)

**Unique**	**29 (26)**

There is high genetic diversity in Kawempe Municipality; the HGDI [19] was 0.996, which is very high and comparable to that of Mulenga et al (0.988) in Ndola, Zambia [22], an urban setting in an endemic country similar to Uganda. The high genetic diversity of MTB strains in Kawempe community could be a consequence of reactivation of latent MTB infection, increased human population/global travel and diversity in host genetics [23].

### MIRU-VNTR patterns

**Figure 1:**
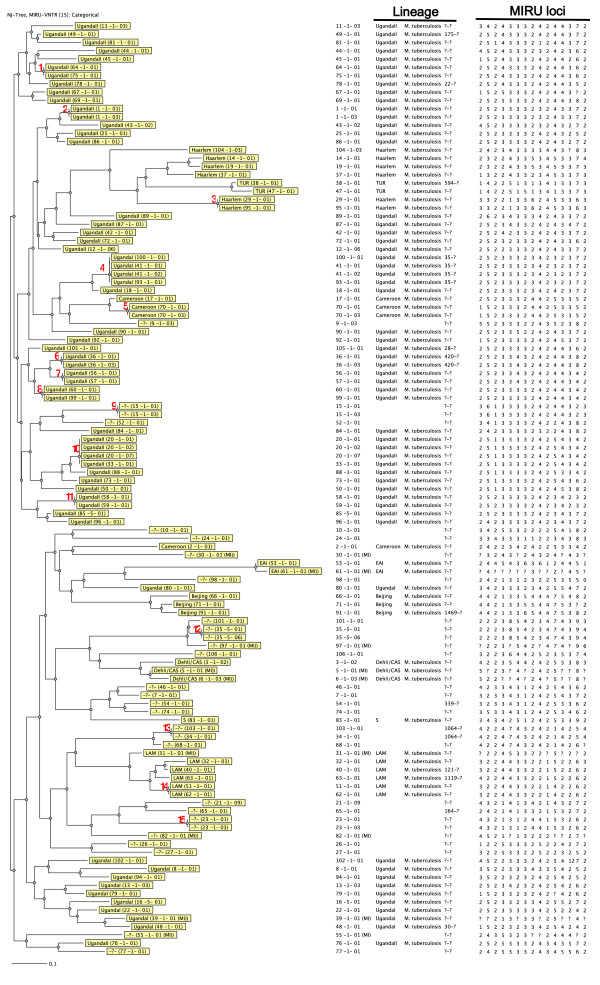
**Neighbor joining dendrogram showing clustering among the genotyped MTB strains**. The 15 clusters are indicated with a numerical brown font. The order of MIRU loci is as follows, left to right: 424, 577, 580, 802, 960, 1644, 1955, 2163b, 2165, 2401, 2996, 3192, 3690, 4952 and 4150.

There was high diversity of MIRU-VNTR patterns among the characterized isolates; a total of 94 distinct patterns were identified, which included 15 clusters each with two to four isolates. In total 34 (30%) isolates clustered while 79 (70%) had unique patterns (see Figure 1). The clustering rate was 17%, implying that the minimum estimate of disease related to recent transmission was 17% (see methods). This is low considering the high population density, endemicity, poor housing and HIV/AIDS prevalence in Kawempe municipality [4], which are risk factors for TB transmission. Similar to the Casablanca study [18], most disease in this community could be due to reactivation of MTB infection rather than recent transmission. Furthermore, the clustering rate was low in comparison with an earlier study in Rubaga municipality Kampala, Uganda [24], in which a high clustering rate was reported using *IS6110*-RFLP genotyping. The differences in clustering rates between the current and former study could be attributed to the high discriminatory power of the 15 loci MIRU-VNTR genotyping panel (HGDI of 0.996).

If true, the possible impact of the presence of low transmission rate, and the implication that most disease in this community could be due to reactivation of MTB infection could be highly influential with regards to infection control measures and disease management. Probably in future, treatment of latent MTB infections should be considered as a control strategy in high endemic areas as it is in industrialized settings. Nevertheless, TB transmission could still be high in Kawempe municipality; the fact that this study only looked at one culture sample per patient, more samples from more households (and more genotyping methods done on each sample) will be needed for conclusive findings.

Six clusters (2, 5, 6, 9, 12 and 15, see Figure 1), each with two isolates (12 isolates in total) involved members of the same household, implying possible household transmission. Two clusters (4 and 10, see Figure 1), each with four isolates, involved participants within the same household and those outside the household (implying a common strain among them). Seven clusters (1, 7, 8, 11, 13, 14 and 15, see Figure 1), each with two isolates, were from epidemiologically unlinked participants. Recent transmission of PTB was found in only 17 patients (15%) who had epidemiologically linked strains. Overall, 17 households had patients with similar strains but the corresponding cultures for 10 index cases were missing. Eight households had similar MTB strains for both index and contact cases, indicating possible transmission (see Table 2). However, household transmission could be higher than we are reporting if the index cases for the other contacts were available.

**Table 2:** MTB transmission patterns in households

			MIRU-VNTR locus^a^														
			
	PT ID	Lineage^b^	424	577	580	802	960	1644	1955	2163b	2165	2401	2996	3192	3690	4052	4156
**(J)**	1 -1- 01	Uganda II	2	5	2	3	3	3	3	2	3	2	4	3	3	7	2
	1 -1- 03	Uganda II	2	5	2	3	3	3	3	2	3	2	4	3	3	7	2
**(K)**	15 -1- 01		3	6	1	3	3	3	3	2	4	2	4	4	3	2	3
	15 -1 - 03		3	6	1	3	3	3	3	2	4	2	4	4	3	2	3
**(L)**	20 -1- 01	Uganda II	2	5	1	3	3	3	3	2	4	2	5	4	3	4	2
	20 -1- 02	Uganda II	2	5	1	3	3	3	3	2	4	2	5	4	3	4	2
	20 -1- 07	Uganda II	2	5	1	3	3	3	3	2	4	2	5	4	3	4	2
	33-1-01	Uganda II	2	5	1	3	3	3	3	2	4	2	5	4	3	4	2
**(M)**	23 -1- 01		4	2	2	1	3	1	2	2	4	1	5	3	2	7	2
	23 -1- 03		4	3	2	1	3	1	2	2	4	1	5	3	2	7	2
**(N)**	35 -5- 01		2	2	2	3	8	5	4	2	3	4	7	4	3	9	4
	35 -5- 06		2	2	2	3	8	5	4	2	3	4	7	4	3	9	4
																	
**(O)**	36-1-01	Uganda II	3	4	2	3	2	3	2	3	4	2	4	2	5	8	4
	36-1-03	Uganda II	3	4	2	3	2	3	2	3	4	2	4	2	5	8	4
**(P)**	41 -1- 01	Uganda I	2	5	2	3	3	3	2	3	4	2	3	2	3	7	2
	41 -1- 02	Uganda I	2	5	2	3	3	3	2	3	4	2	3	2	3	7	2
	93-1-01	Uganda I	2	5	2	3	3	3	2	3	4	2	3	2	3	7	2
	100-1-01	Uganda I	2	5	2	3	3	3	2	3	4	2	3	2	3	7	2
**(Q)**	70 -1- 01	Cameroon	1	5	2	3	3	3	2	4	4	2	5	3	3	5	2
	70 -1- 03	Cameroon	1	5	2	3	3	3	2	4	4	2	5	3	3	5	2
																	

**^a^**The numerical figures refer to the number of alleles per PCR-amplified MIRU-VNTR locus [8,17].

**^b^**Blanks for **K, M **and **N **indicate unique strains i.e., those without matching strain in the database. Letters in parenthesis [**(J), (K), (L), (M), (N), (O), (P) **and **(Q)**] represent households.

Figures under **PTID **(Patient Identification) column refer to the index cases (ending with 1, e.g., 1-1-01) and contact cases (ending with a numerical value > 1, e.g., 1-1-03). For (L) and (P), a similar strain was transmitted to patients in other households.

## Conclusion

MIRU-VNTR genotyping revealed low clustering and high diversity of MTB strains in Kawempe municipality and confirmed earlier reports that MTB strain "Uganda genotype" is the predominant lineage in Kampala, Uganda. MIRU-VNTR typing with a panel of 15 loci is applicable in a Ugandan setting, and can unravel clonally complex strains into individual strains. A nation-wide study to determine the full spectrum of circulating MTB strains in Uganda will be helpful.

## Abbreviations

MIRU-VNTR: mycobacterial interspersed repetitive units - variable number of tandem repeats; HGDI: Hunter Gaston Discriminatory Index.

## Competing interests

The authors declare that they have no competing interests.

## Authors' contributions

LN and KRD carried out the experimental procedures. LN, DPK, FAK and PRO analyzed the data. DPK wrote the manuscript. KRD, CCW and MLJ conceived the study, participated in its design and coordination and proofread the manuscript. All authors read and approved the final manuscript.

## Supplementary Material

Additional file 1 **MTB strain types with reference to the MIRU-VNTR*plus *database**. Highlighted yellow are the households where transmission was predicted. Patients with mixed infections are highlighted in grey. **^a^PTID **(Patient Identification); index cases end with 1 (e.g., 1-1-01) while contact cases end with a numerical value > 1 (e.g., 1-1-03). **^b^**Blanks refer to unique strains i.e., those without a matching strain in the database.**^c^**The numerical figures refer to the number of alleles per PCR-amplified MIRU-VNTR locus [8,17]. Click here for file
